# Exploring C-peptide loss in type 1 diabetes using growth curve analysis

**DOI:** 10.1371/journal.pone.0199635

**Published:** 2018-07-03

**Authors:** Rachel E. J. Besser, Johnny Ludvigsson, Peter C. Hindmarsh, Tim J. Cole

**Affiliations:** 1 Genetics and Epigenetics in Health and Disease, University College London, Great Ormond Street Institute of Child Health, London, United Kingdom; 2 Department of Clinical and Experimental Medicine, Division of Pediatrics, Medical Faculty, Linköping University, Linköping, Sweden; 3 Developmental Endocrinology Research Group, University College London Great Ormond Street Institute of Child Health, London, United Kingdom; 4 Population, Policy and Practice Programme, University College London Great Ormond Street Institute of Child Health, London, United Kingdom; La Jolla Institute for Allergy and Immunology, UNITED STATES

## Abstract

**Objectives:**

C-peptide (CP) loss in type 1 diabetes (T1D) is highly variable, and factors influencing it are poorly understood. We modelled CP values in T1D patients from diagnosis for up to 6 years, treating the serial data as growth curves plotted against time since diagnosis. The aims were to summarise the pattern of CP loss (i.e. growth curve shape) in individual patients in simple terms, and to identify baseline characteristics that predict this pattern in individuals.

**Materials and methods:**

Between 1976 and 2011, 442 T1D patients initially aged <18y underwent 120-minute mixed meal tolerance tests (MMTT) to calculate area under the curve (AUC) CP, at 3, 9, 18, 30, 48 and 72 months after diagnosis (n = 1537). The data were analysed using the novel SITAR mixed effects growth curve model (SuperImposition by Translation And Rotation). It fits a mean AUC growth curve, but also allows the curve’s mean level and rate of fall to vary between individuals so as to best fit the individual patient curves. These curve adjustments define individual curve shape.

**Results:**

The square root (√) AUC scale provided the best fit. The mean levels and rates of fall for individuals were normally distributed and uncorrelated with each other. Age at diagnosis and √AUC at 3 months strongly predicted the patient-specific mean levels, while younger age at diagnosis (p<0.0001) and the 120-minute CP value of the 3-month MMTT (p = 0.002) predicted the patient-specific rates of fall.

**Conclusions:**

SITAR growth curve analysis is a useful tool to assess CP loss in type 1 diabetes, explaining patient differences in terms of their mean level and rate of fall. A definition of rapid CP loss could be based on a quantile of the rate of fall distribution, allowing better understanding of factors determining CP loss and stratification of patients into targeted therapies.

## Introduction

Area under the curve C-peptide (AUC CP) based on a mixed meal tolerance test (MMTT) is the gold standard measure of beta cell loss in Type 1 diabetes (T1D) [1–3]. CP typically rises in the first weeks to months after diagnosis and then falls over time. Both the starting level of CP, reflecting beta cell reserve, and its rate of decline, indicating disease progression, vary considerably between patients [1–7]. An individual’s disease course can be visualised by plotting their CP against time since diagnosis until CP becomes undetectable.

For future intervention studies it would be useful to be able to predict disease course in individual patients from factors available soon after diagnosis. Age is the strongest predictor of beta cell loss; a younger age at diagnosis is associated with a lower starting beta cell reserve [8–10] as well as a more rapid rate of loss [11, 12]. Diabetic ketoacidosis is also unsurprisingly associated with poor beta cell recovery; perhaps as a marker of low beta cell mass [13, 14]. Other factors which may be predictive include high titer multiple islet auto antibodies, intensive insulin treatment, genetic susceptibility (DR3/DR4-DQ8 genotype), and body mass index [15–23]. However other drivers are unknown making it difficult to predict beta cell loss in the individual patient.

An efficient way to identify potential predictive factors would be to retrospectively analyse serial CP data, distinguishing between predictors of starting CP and the rate of CP decline. However this is complicated by the fact that these two outcomes are inevitably correlated, whereas the factors predicting them may be less so. The challenge is how best to analyse the data so as to identify predictive factors for these two outcomes.

The plot of CP versus time in individuals can be viewed as a form of growth curve. As such it is amenable to statistical methods of growth curve analysis, in particular a novel method called SITAR (SuperImposition by Translation And Rotation), first described in 2010 [24]. The method summarises a set of growth curves (e.g. CP versus time in a group of patients) as a mean growth curve, plus a set of up to three patient-specific adjustments which modify the mean curve to match the individual patient curves.

SITAR has been used in a number of biological settings, notably height in puberty, where its three subject-specific adjustments explain over 99% of the between-subject variability in height growth [25–27]. AUC CP falling in T1D is analogous to height rising in puberty, and just as a height curve can be estimated, we hypothesise so too can a curve for AUC CP.

The objective of the paper is to apply the SITAR model to “growth” curves of CP plotted against time since diagnosis in a cohort of T1D patients aged <18 years at diagnosis and followed for up to 6 years. Specific aims are to 1) illustrate the variation in beta cell loss over time in individual patients; 2) show how the SITAR model summarises the individual CP growth curves in terms of two patient-specific parameters: their mean level and rate of fall; and 3) identify baseline factors and features of the mixed meal tolerance test (MMTT) curve taken at 3 months that predict the mean level and rate of fall in individuals.

## Materials and methods

### Patients studied

We studied 442 patients diagnosed with type 1 diabetes under 18 years of age, as described previously [18]. They were part of a unique “natural history” cohort of patients attending the Pediatric Diabetes clinic at University Hospital, Linköping, Sweden between July 1976 and March 2011. Over 90% of patients aged over 7 years participated in the study, as reported previously [28].

### Blood sampling

Patients had a random CP measured prior to the first insulin injection, and routine MMTTs were undertaken at 3, 9, 18, 30, 48 and 72 months following diagnosis. The MMTTs were performed under standard conditions with blood sampling every 30 minutes over 150 minutes, as previously described [18]. MMTTs were stopped once peak CP had fallen below 0.03 nmol/L (the detection limit of the assay), on two consecutive occasions.

### Ethical considerations

Ethical approval was gained through the Research Ethics Committee of the Faculty of Health Sciences, Linköping University, and has been described previously [18]. The parents/ guardians and older children had given informed consent to participate in the Mixed Meal Tolerance tests and for use of these results for research. Furthermore, all data were fully anonymized before they were used in the analyses.

### Laboratory methods

During the 35 year study period, CP was analyzed by three different assays, and samples were stored at -20°C until analysis, as described previously [18]. CP was measured before June 2000 by radioimmunoassay [29]; from June 2000 to September 2004 by enzyme-linked immunosorbent assay (DRG Diagnostics, Marburg, Germany), and from October 2004 by fluoroimmunoassay (AutoDELFIA C-peptide kit; Wallac) with an associated software program (1224 MultiCalc; Wallac) for calculation.

### Outcome measure

Mean CP was measured as AUC in the first 120 minutes of the MMTT (AUC 120), calculated by the trapezoid rule in units of nmol/L/120min (n = 1537). The analyses were repeated with AUC 150 for the subset of 420 patients who had a 150-minute value (n = 1331), but the results were similar and are not presented.

### SITAR model

The SITAR model is a shape-invariant mixed effects growth curve model, which summarises a set of growth curves, in this case a set of AUC CP ‘growth curves’, with AUC on the y-axis and time since diagnosis on the *x*-axis. The SITAR model estimates the mean curve, and also patient-specific adjustments (or *random effects)* that modify the mean CP curve to match the individual patient curves [24]. The SITAR model assumes that individual curves differ from the mean curve in just two ways–size and intensity–as defined below. The full SITAR model also includes a third effect related to timing, but it is does not apply here.

The subject-specific “size” random effects shift the mean curve up or down the *y*-axis to best match the curves for individuals–this size effect is here termed the “mean level”. The “intensity” random effects stretch or shrink time on the *x*-axis, which alters the slope of the mean curve to best match the slopes for individual curves–this intensity effect is here called the relative rate of fall, or “rate of fall” for short. The two effects (mean level and rate of fall) have mean zero by definition, and positive values of the rate of fall indicate a relatively steep curve, and negative values relatively shallow; the rate of fall is in fractional units and can be multiplied by 100 and viewed as a percentage. If the model fits well, adjusting for the mean level and rate of fall in individuals causes their curves to be superimposed on the mean curve.

The SITAR mean curve used a cubic spline with 3 degrees of freedom. We tested various versions of the model, with both AUC and time on the original, log and square root (√) scales, and chose the model with √AUC versus time that best superimposed the individual curves, identified by minimising the Bayesian Information Criterion (BIC) [30].

We then used multiple linear regression to separately explore predictors of the mean level and rate of fall in individuals, selecting from the following covariates available at the visit 3 months after diagnosis: gender, age of diagnosis, CP prior to first insulin injection, and the six half-hour CP values from the MMTT curve at 3 months, along with the 3-month CP AUCs based on 120 and 150 minutes. The CP and AUC values were tested both in original and square root units, to match mean √AUC, and interactions between age, gender and AUC were tested for.

## Results

The cohort of 442 patients was included in the analysis, with 54% (240) male, and mean (SD) age of diagnosis 11.0 (3.5) years (see Table A in S1 File).

### Variation in beta cell loss over time since diagnosis

Excluding two obvious outliers, a total of 1537 MMTTs were taken at 3, 9, 18, 30, 48 and 72 months following diagnosis; the numbers of MMTTs and the median AUC both fell over time (Table A in S1 File). The numbers of patients undergoing one to six MMTTs were respectively 58, 70, 80, 106, 93 and 35.

**Fig 1:**
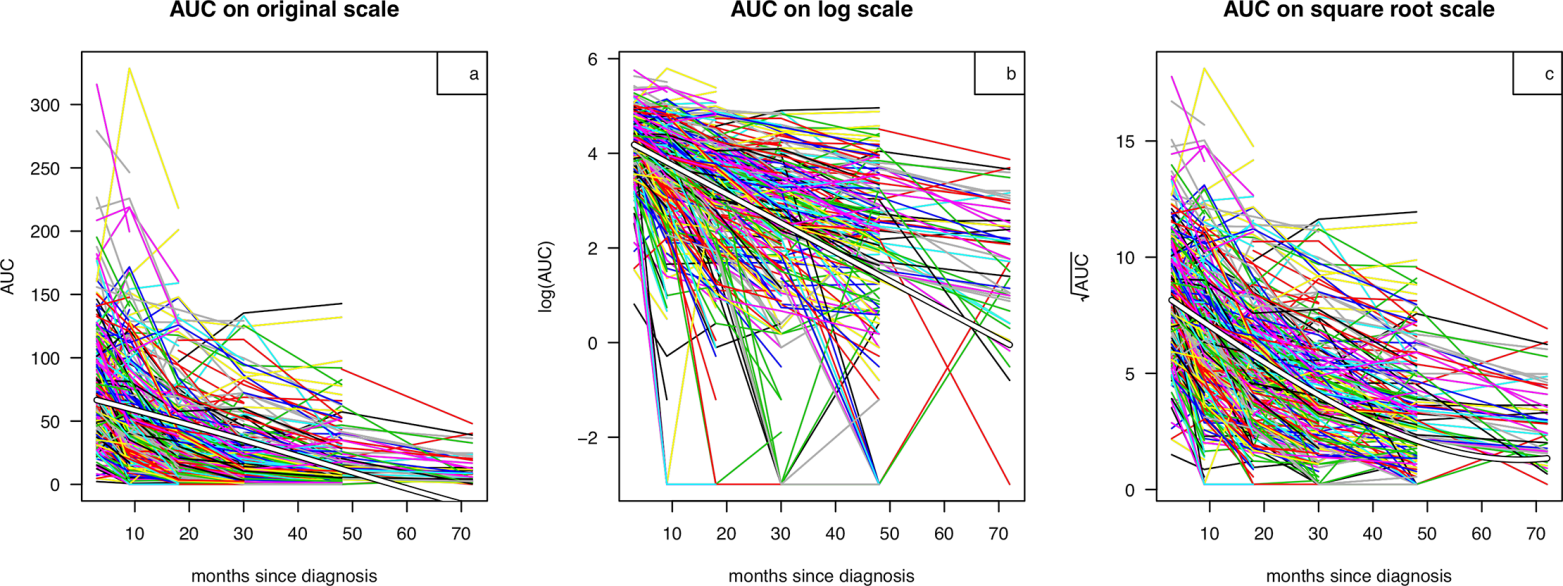
**Growth curves of AUC C-peptide versus time since diagnosis in individual patients (coloured lines), with AUC plotted on the (a) original, (b) logarithmic and (c) square root scale.** The corresponding mean curves (white) from the fitted SITAR models are shown superimposed.

Fig 1 presents the individual growth curves of AUC plotted against time since diagnosis, color-coded by patient, with AUC on the original, log and square root transformed scales respectively. The corresponding mean curves as fitted by SITAR are also shown superimposed. On the AUC scale the fitted curve is close to linear and crosses zero at 58 months, indicating a very poor fit. Similarly on the log scale the fitted curve is close to linear, while on the square root scale the curve is appreciably nonlinear. Fig A in S1 File compares the three fitted curves back-transformed to the AUC scale, showing that the log and square root curves are broadly similar in shape. The square root scale provides the best fit (BIC = 13488, 14287 and 14850 respectively on the square root, log and original scales), and it explains 79% of the variance.

By inspection, the model on the original scale focuses on the upper tail of the AUC distribution, whereas on the log scale the lower tail dominates. The square root scale provides a compromise that takes into account both tails of the AUC distribution.

### SITAR-adjusted model for beta cell loss over time: Mean level and rate of fall

**Fig 2:**
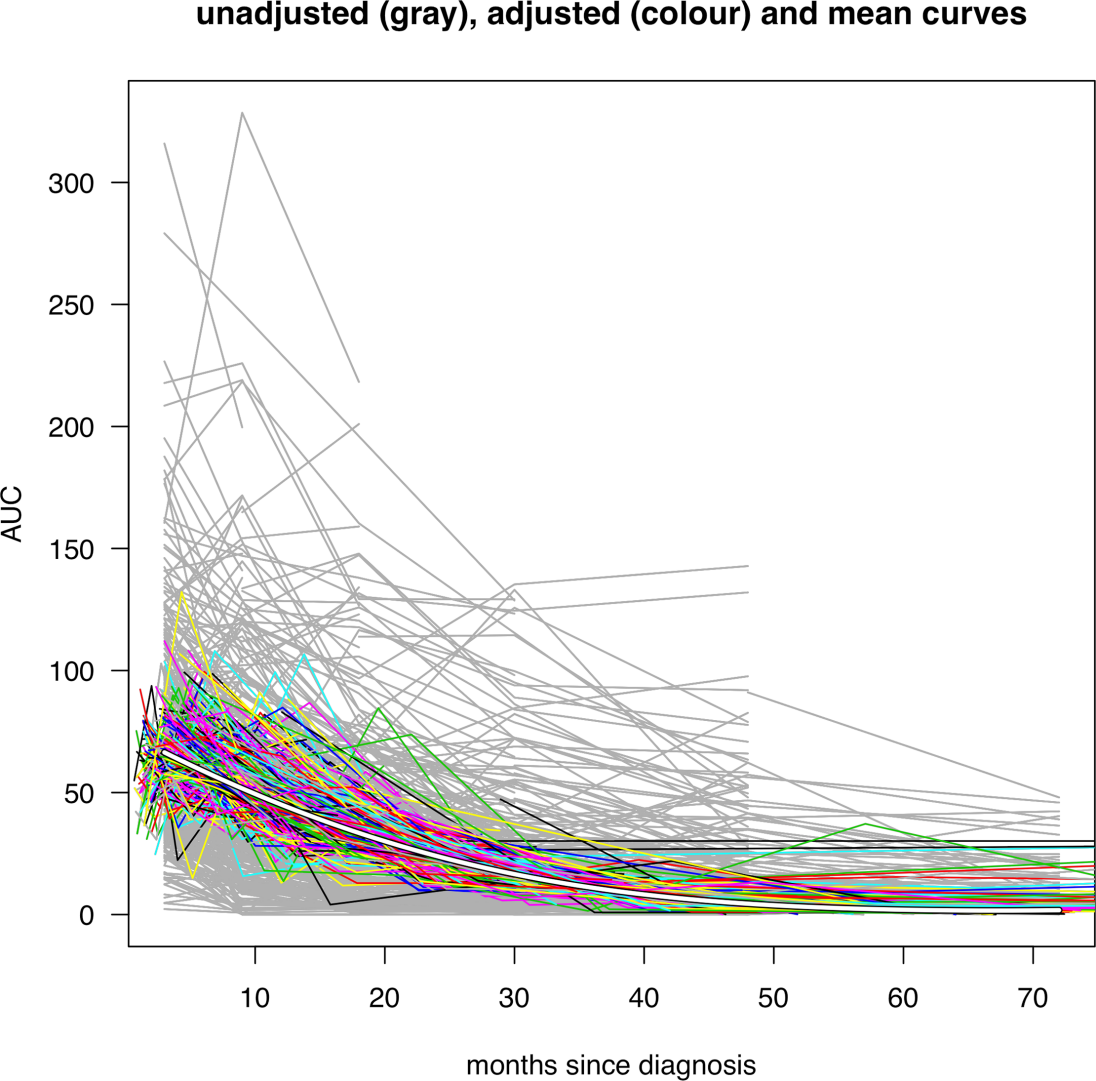
Growth curves of AUC C-peptide for the √AUC model shown unadjusted (gray) and SITAR adjusted (colour), with the mean curve (white).

**Fig 3:**
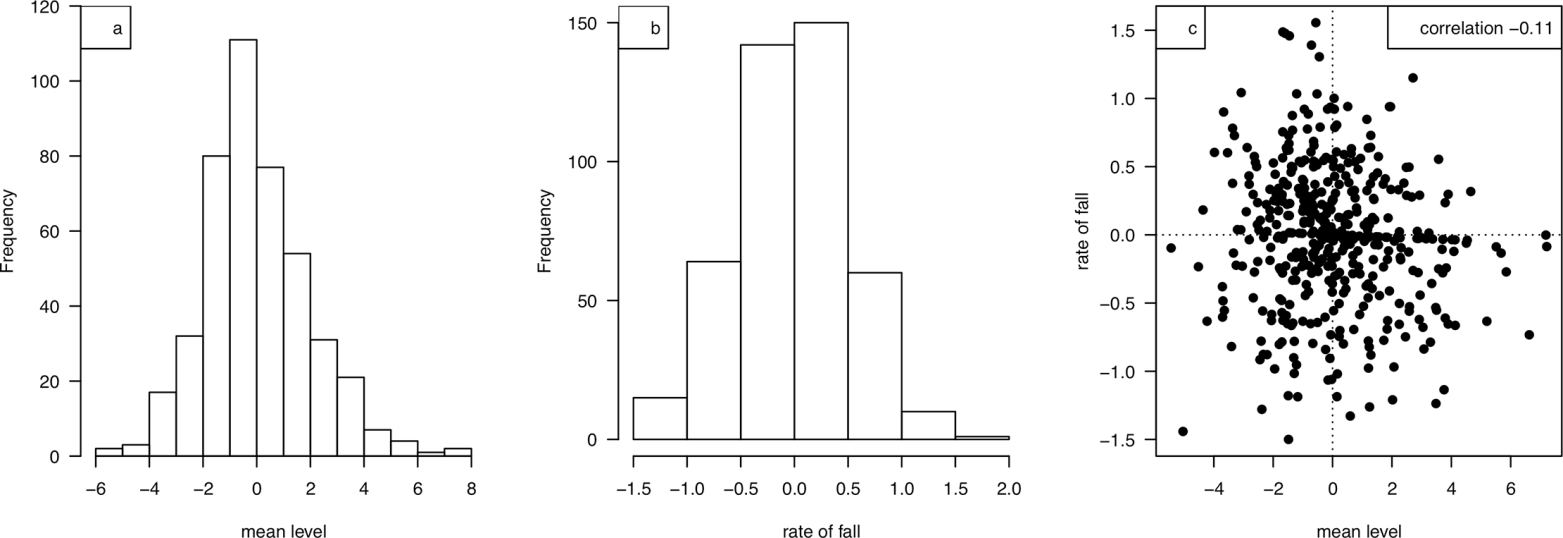
Histograms of the SITAR random effects (a) mean level and (b) rate of fall, and (c) scatterplot of rate of fall versus mean level.

Fig 2 shows the individual growth curves before and after SITAR adjustment using the square root model, with the mean curve superimposed. The model summarises the individual curves in terms of the two patient-specific parameters, mean level and rate of fall. Both are reasonably normally distributed, and uncorrelated with each other (Fig 3). The standard deviations for the two parameters are 2.2 and 0.68 respectively.

### Which features of the 3-month MMTT predict mean level and rate of fall?

#### Mean level

Most (91%) of the variance in the mean level is explained by age at diagnosis and √AUC at 3 months (Table B in S1 File). The mean level is unsurprisingly higher in patients diagnosed at an older age (p<0.0001) and in females (p = 0.0004). The mean level also correlates very strongly with the 120 min AUC from the 3-month MMTT curve, and adding it to the multiple regression model makes gender insignificant. The 120 minute AUC fits even better when square root transformed to match the mean √AUC transformation (p = 0.001).

#### Rate of fall

Just 5.7% of the variance in the rate of fall is explained (Table C in S1 File), the rate of fall being lower in those diagnosed at an older age (p<0.0001), but unrelated to gender. The coefficient of -0.042 for age of diagnosis indicates that being diagnosed 1 year older reduces the predicted rate of fall by 4.2%.

The 120-minute CP value at 3 months is strongly positively correlated with the rate of fall, and more so with CP square root transformed (p = 0.002). For CP at 120 minutes (median 0.60, IQR 0.38 to 0.95 nmol/L), where the regression coefficient for √CP is 0.37, a shift upwards from the median to the upper quartile (i.e. √0.95 - √0.60 = 0.2 on the square root scale) increases the predicted rate of CP fall by 0.2 x 0.37 = 0.073 or 7.3%.

## Discussion

We present a novel tool, the SITAR model, to define beta cell loss based on serial AUC C-peptide data.

### What is the optimal method of assessing AUC CP?

Conventional models to assess beta cell decline use CP on the original or logarithmic AUC scale. Our results show that the square root scale is better, as proposed by Lachin *et al* [31] based on a different argument. Whilst the data presented in this way are not user-friendly, they can be back-transformed to the AUC scale for presentation purposes. Our approach also differs from conventional models that hold the time axis constant, as SITAR includes a patient-specific time adjustment.

### What affects C-peptide loss?

We have shown that the pattern of CP loss in individuals (as summarized by mean level and the rate of fall) is normally distributed.

The rate of fall is more rapid in younger patients, in keeping with some but not all studies. Recently, an analysis of TrialNet data incorporating the placebo arms of two trials and the intervention and placebo arms of a third trial found that CP increased with age, but there was no difference in the rate of fall between adults (aged ≥21years) and pediatric patients [11]. Our study cohort was younger (mean age (SD) 11.0 (3.5) vs 18.1 (8.8) years) and studied over a longer duration of diabetes (72 vs 24 months).

Many patients continue to secrete CP long past diagnosis [7, 32, 33]. In both our cohort and TrialNet, 52–66% patients at 24–30 months had CP >0.2 nmol/L, a clinically significant concentration [15]. Further exploration of TrialNet data extending out to 4 years post diagnosis, found that up to 31% patients had C-peptide >0.2nmol/L [6]. Our study assesses patients up to 72 months post-diagnosis, by which time the majority (78%) are CP negative [18], and this may allow us to better identify factors affecting CP loss. The lack of correlation between mean level and the rate of fall, in keeping with findings from TrialNet, is useful, as the two factors can be considered independent, and drivers of each may be different.

### Can the 3-month MMTT be used to predict mean level and rate of fall?

The ability to find a marker soon after diagnosis to predict future C-peptide decline would be beneficial, as it would offer the potential to stratify patients into interventions based on individualized risk. The √AUC at 3 months was perhaps unsurprisingly a reliable predictor of mean √AUC (91% of variance explained), as serial AUC values are strongly correlated. This could be useful, as intervention trials frequently use CP 0.2 nmol/L as a secondary outcome.

In contrast, the 3-month MMTT data predicted the rate of CP fall less well (5.7% of variance explained). Even so, the 120-minute CP value at 3 months was highly significantly positively correlated with the rate of CP fall, suggesting that an extended right tail of the 3-month MMTT curve is associated with more rapid subsequent CP decline, but the association was too weak to reliably predict it in individual patients.

It would be important for these results to be replicated in another large dataset, along with other factors such as HLA genotype and islet antibody titer, which could add to the model and better explain the variability in the rate of fall.

### Implications

The finding that the rate of fall is normally distributed has major implications, as its distribution could be used as the basis for a definition of beta cell loss. The choice of cut-off would be arbitrary, but one possibility would be a suitably low quantile from the distribution. By defining beta cell loss in this way, rapid and slow progressors could be identified as those falling in the two tails of the distribution. This would provide a major platform for testing hypotheses of disease mechanism, and in turn could explain disease heterogeneity, and improve interpretation of data from intervention trials.

### Strengths and limitations

The advantage of using these data is that they are truly a natural history dataset, unlike other series that include placebo arms of intervention trials, and so there is no ascertainment bias. In this dataset, patients were followed until they become CP negative (peak CP <0.03nmol/L) on two consecutive occasions up to 72 months after diagnosis. To our knowledge, this is the longest prospective study of such patients.

The model has been fitted to data starting 3 months after diagnosis. Whilst this time period should represent metabolic stability and the time of peak beta cell recovery, CP decline may have already begun in some patients.

We have only been able to assess age and gender in this model. Other important factors that may affect CP loss were not assessed, such as antibody status (type and titer), genetic susceptibility (e.g. HLA type), body mass index and ethnicity. We have also not tested whether the rate of fall predicts HbA1c or longer-term outcomes such as microvascular complications.

During the study period (1976–2011) two different meal stimuli and three different CP assays were used. The CP values obtained after 2000 were slightly higher than before, which would potentially underestimate the rate of beta cell decline.

The model is also biased towards patients with preserved beta cell function, as testing stops once peak CP is below 0.03 nmol/L (the detection limit of the assay) on two consecutive occasions.

### Future studies

The model needs validation in other large datasets, and should be extended to adult patients. It could be used to test factors (e.g. genetic susceptibility, antibody status, treatment regimen, ethnicity) associated with the most extreme rates of CP loss, as well as factors associated with high/low mean level and high/low rate of fall.

## Conclusions

We have demonstrated that the SITAR model fits serial CP data well and is a valid method of assessing beta cell loss in new onset type 1 diabetes, by adjusting for both mean level and rate of fall. The model may become useful in predicting which newly diagnosed type 1 diabetes patients will develop rapid beta cell loss, allowing stratification of patients into targeted therapies.

## Supporting information

S1 File Containing Tables A, B and C and Fig A.
(ZIP)
