# Supplementary material for: Exploring C-peptide loss in type 1 diabetes using growth curve analysis
Source: PLoS One. 2018 Jul 3;13(7):e0199635. doi: 10.1371/journal.pone.0199635 (PMC6029769; doi:10.1371/journal.pone.0199635)

# Online-Only Supplemental Materials

**Supplemental Tables: 3**

**Supplemental Figures: 1**

**LEGENDS TO SUPPLEMENTAL TABLES AND FIGURES**

Supplemental Table A. Summary of 442 patients.

Supplemental Table B. Predictors of mean √AUC at 3 months.

Supplemental Table C. Predictors of rate of CP fall at 3 months.

Supplemental Figure A. The three mean curves from Figure 1 replotted on the AUC scale, where the log and square root curves are similar in shape.

# Supplemental Table A. Summary of 442 patients.

|  | N | Mean (SD) | Range |
| --- | --- | --- | --- |
| Age of diagnosis (years) | 442 | 11.0 (3.5) | (1.1, 17.6) |
|  |  | Median (IQR) | Range |
| C-peptide at baseline (nmol/L) | 375 | 0.22 (0.14, 0.35) | (0.01, 1.6) |
| AUC C-peptide at 3 months | 363 | 61 (39, 95) | (2.2, 316) |
| AUC C-peptide at 9 months | 337 | 41 (23, 69) | (0, 328) |
| AUC C-peptide at 18 months | 314 | 25 (9.9, 47) | (0, 218) |
| AUC C-peptide at 36 months | 269 | 15 (4.8, 33) | (0, 135) |
| AUC C-peptide at 48 months | 193 | 10 (3.2, 25) | (0, 143) |
| AUC C-peptide at 72 months | 61 | 8.0 (2.7, 17) | (0, 48) |

# Supplemental Table B. Predictors of mean level at 3 months.

|  | Regression coefficient | Standard error | T | P |
| --- | --- | --- | --- | --- |
| Age of diagnosis | 0.037 | 0.011 | 3.3 | 0.001 |
| 120-minute √AUC at 3 months | 0.76 | 0.01 | 52 | <0.0001 |

Residual standard error: 0.62 on 323 degrees of freedom

Adjusted R-squared: 0.91

# Supplemental Table C. Predictors of rate of fall at 3 months.

|  | Regression coefficient | Standard error | T | P |
| --- | --- | --- | --- | --- |
| Age of diagnosis | -0.042 | 0.010 | -4.4 | <0.0001 |
| 120-minute √CP at 3 months | 0.37 | 0.12 | 3.1 | 0.002 |

Residual standard error: 0.53 on 323 degrees of freedom

Adjusted R-squared: 0.057

**Supplemental Figure A. The three mean curves from Figure 1 replotted on the AUC scale, where the log and square root curves are similar in shape.**


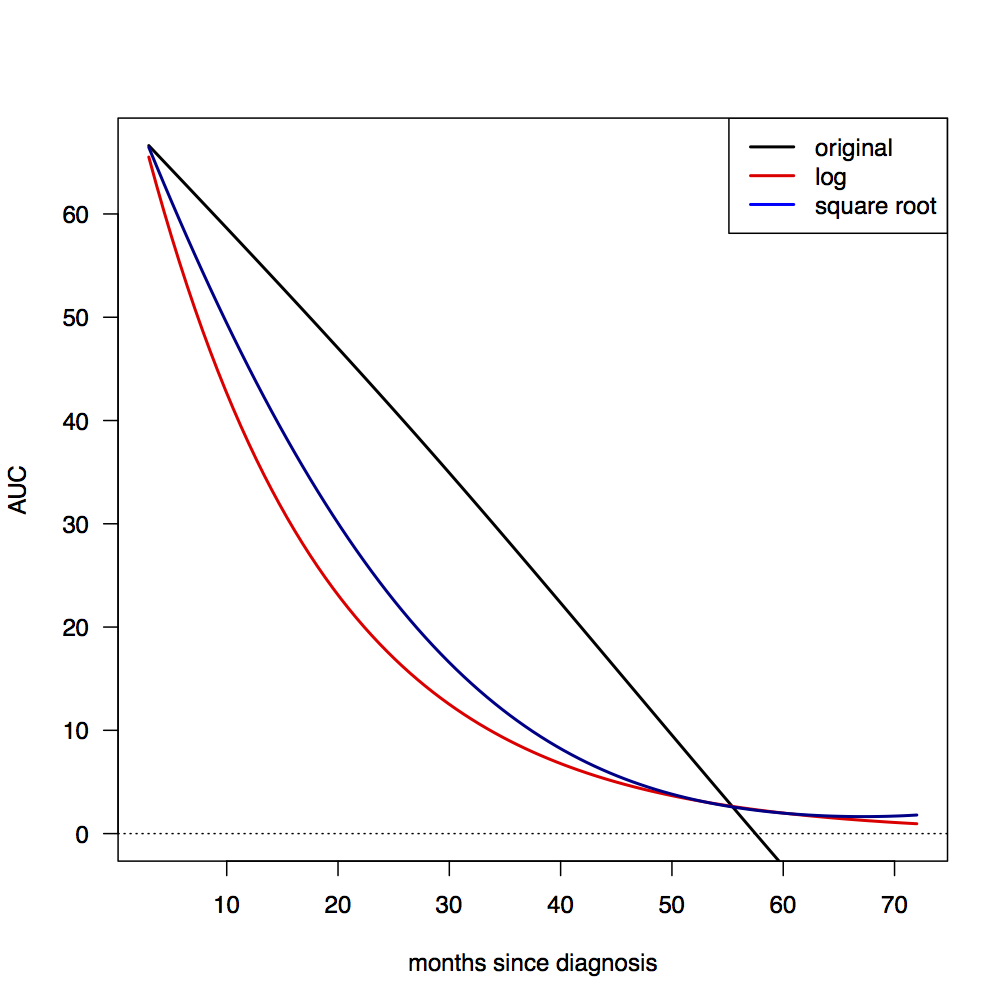

Supplement: S1 File — Containing Tables A, B and C and Fig A. (ZIP) [file pone.0199635.s001.zip › Online-only Supplemetal Materials.docx]
